# Atypical Acute Ischemic Choriocapillaritis: A Case Report

**DOI:** 10.1002/ccr3.9646

**Published:** 2024-12-16

**Authors:** Mehrdad Motamed Shariati, Nasser Shoeibi, Mariye Yaghoubi

**Affiliations:** ^1^ Eye Research Center Mashhad University of Medical Sciences Mashhad Iran

**Keywords:** acute ischemic choriocapillaritis, acute posterior multifocal placoid pigment epitheliopathy, immune‐mediated choroiditis, placoid, primary choriocapillaropathies

## Abstract

Optical coherence tomography angiography is a valuable tool for evaluating acute ischemic choriocapillaritis. Tuberculosis and syphilis are the main secondary differentials..

## 1 Introduction

The choriocapillaris is a specialized layer of the choroid, organized in a distinctive lobular pattern where each lobule is supplied by a single arteriole and drained by a venule. This segmentation allows for localized ischemia, as disruptions in blood flow affect individual lobules while adjacent regions remain unaffected. The choriocapillaris contains fenestrated capillaries that facilitate nutrient exchange between the choroid and the outer retina, which includes the retinal pigment epithelium (RPE) and photoreceptors. Given its crucial role in retinal metabolism, ischemia in the choriocapillaris can lead to significant visual impairment, as seen in conditions like acute ischemic choriocapillaritis. Angiographic techniques, such as indocyanine green angiography (ICGA) and optical coherence tomography angiography (OCTA), have played a crucial role in identifying choroidal perfusion abnormalities. OCTA allows for a detailed assessment of the choroidal circulation due to its ability to penetrate the RPE, providing insights into choroidal blood flow and ischemic changes [1].

Primary inflammatory choriocapillaropathies (PICCPs) are defined as the disruption of choriocapillaris perfusion, which includes a spectrum of mild end‐capillary involvement as in multiple evanescent white dot syndrome (MEWDS) to more severe proximal capillary non‐perfusion as in idiopathic multifocal choroiditis (MFC), acute posterior multifocal placoid pigment epitheliopathy/acute multifocal ischemic choriocapillaritis (APMPPE/AMIC), and serpiginous choroidopathy (SC) [1]. The origin of the PICCPs remains unknown.

The angiographic pattern and clinical course of the disease depend on the level of involvement (capillaries vs. arterioles), its severity, and the reversibility of the vaso‐occlusive process [2]. In severe cases, RPE and photoreceptors cannot survive, which leads to RPE and outer retinal atrophy and permanent visual loss [3].

This report aims to introduce a young man with acute ischemic choriocapillaritis 2 weeks after an upper respiratory tract infection.

## 2 Case History and Examination

**FIGURE 1:**
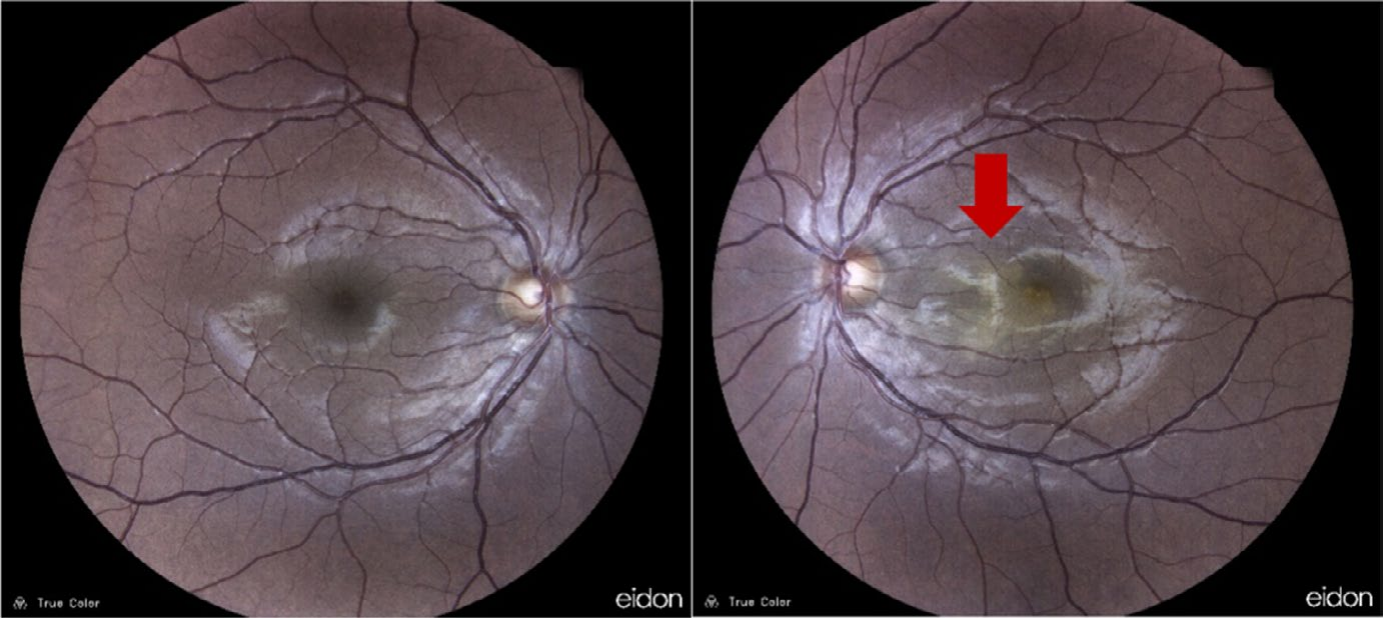
Fundus photographs showed an area of paleness with a geographic pattern at the papillomacular bundle in the left eye (red arrow).

A 34‐year‐old male patient presented to us with acute painless visual loss of his left eye (LE) from 3 days ago. The best‐corrected distance visual acuity (BCDVA) was 10/10 and 3/10 for the right eye (RE) and LE, respectively. The ocular movement was normal in both eyes. Anterior segment examination was unremarkable for both eyes, with no evidence of inflammation or other abnormalities. Fundus examination of the LE showed an area of paleness with a geographic pattern at the papillomacular bundle with the involvement of the nasal foveal region with an estimated size of one disc area (Figure 1). Fundus examination of the RE was unremarkable, with no signs of ischemia or other pathology.

## 3 Methods

**FIGURE 2:**
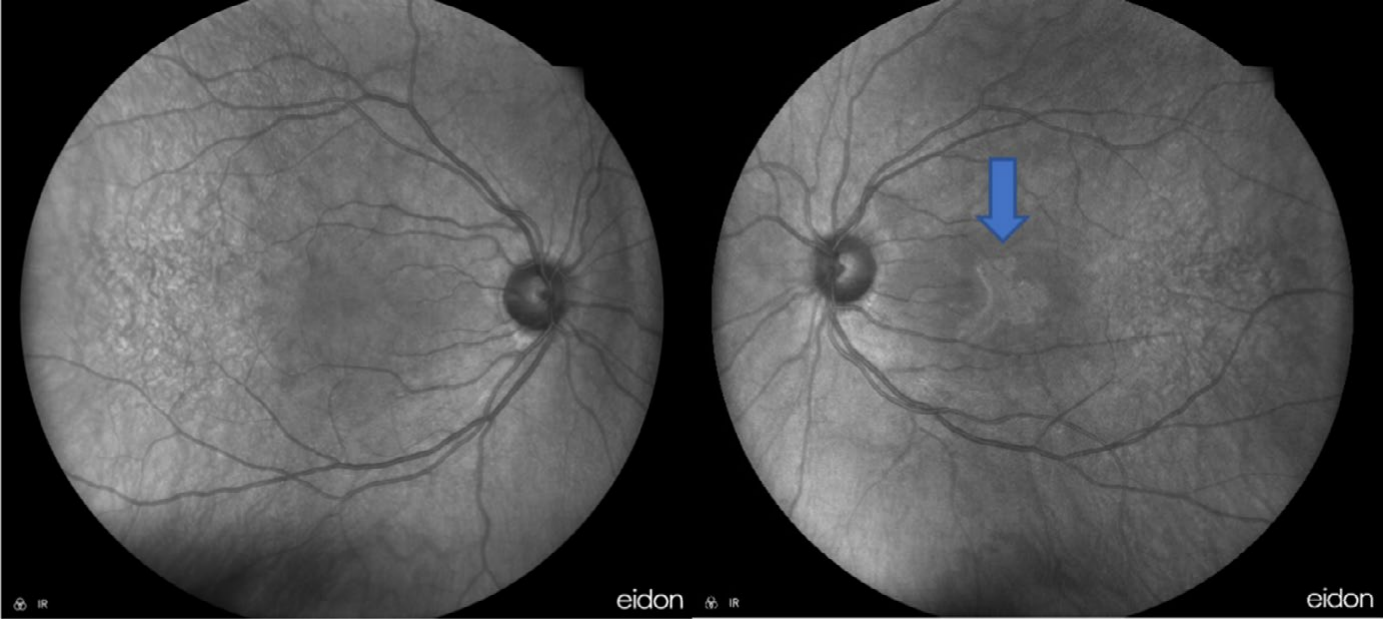
The near‐infrared reflectance images showed a geographic hyperreflective lesion at the left eye's macula (blue arrow).

**FIGURE 3:**
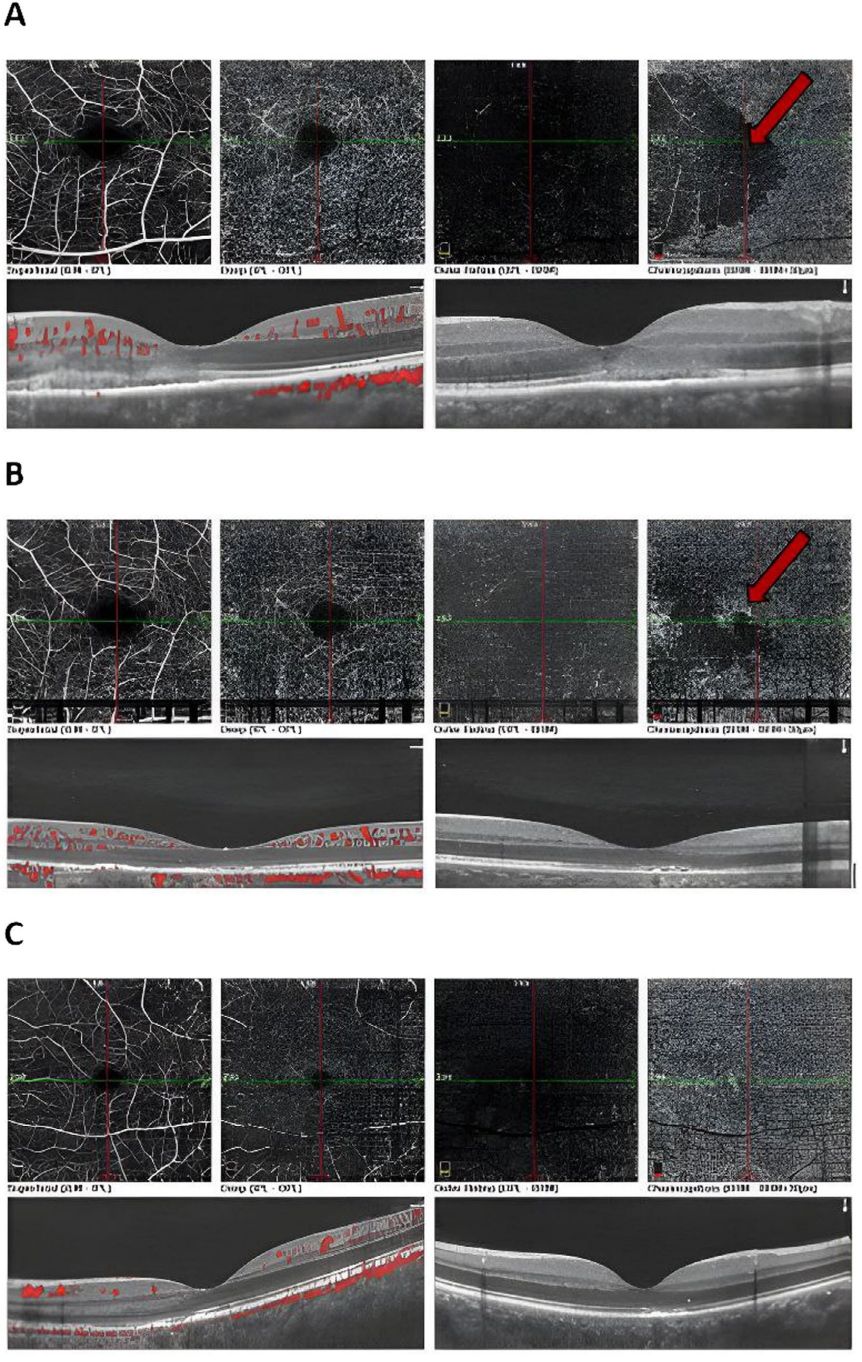
OCTA images of the LE revealed a wedge‐shaped choriocapillaris dropout (red arrow) at the first examination (A), which resolved during the follow‐up period (B, C).

We used multimodal imaging, including near‐infrared reflectance (Heidelberg Eye Explorer version 1.9.13.0, Spectralis Viewing Module 6.5.2.0; Heidelberg Engineering) (Figure 2) and optical coherence tomography angiography (OCTA) (OptoVue Inc., Fremont, CA, USA, software version: 2018,0,0,18) (Figure 3A) for further evaluation. Ellipsoid zone (EZ) discontinuity and choriocapillaris drop‐out were the main OCTA findings at the presentation.

We considered systemic work‐ups to rule out secondary causes of choriocapillaritis, including tuberculosis (TB) and syphilis. Our patient's past medical history was unremarkable. The purified protein derivative (PPD) skin test was negative. Also, there were no remarkable findings in the infectious diseases specialist consultation.

Regarding the suspicion of acute ischemic choriocapillaritis and visual impairment, oral prednisolone was started at a dose of 50 mg/day.

## 4 Results

The follow‐up examination was conducted 1 week later. OCTA image of the choriocapillaris showed a significant improvement in perfusion (Figure 3B). The prednisolone dose was tapered off 2 weeks later. At the last follow‐up visit (3 weeks after the first presentation), the BCDVA improved to 10/10 for the left eye, and capillary dropout was resolved (Figure 3C).

## 5 Conclusion

This case highlights a rare presentation of unilateral and unifocal acute ischemic choriocapillaritis, which responded favorably to corticosteroid therapy. The recovery of choriocapillaris perfusion and visual function demonstrates the potential for reversibility in ischemic choriocapillaritis when timely treatment is initiated. The combination of OCTA and clinical examination was essential for diagnosis, monitoring, and guiding treatment.

## 6 Discussion

Inflammation at the level of the choriocapillaris causes areas of choriocapillaris nonperfusion and ischemic consequences in the choroid and outer retina [1].

PICCPs are categorized as choriocapillaritis with an unknown trigger factor. However, some patients show flu‐like symptoms before the onset of ocular disease. Depending on the level of capillary involvement, PICCPs show a spectrum of signs and symptoms, from mild and reversible damage in MEWDS to irreversible scars in MFC and SC [1]. Patients usually complain of blurred vision and scotomas at the presentation [1, 2].

Multimodal imaging is essential to identify the extension and severity of involvement and help the clinician categorize the disease [4]. We summarized the clinical presentation and imaging findings in different kinds of PICCPs in Table 1.

**TABLE 1:** Clinical, OCT, and OCTA characteristics of primary inflammatory choriocapillaritis [1, 2].

	Gender	Clinical presentation	Laterality	Main clinical findings	OCT findings	OCTA findings
The presented case	Male	Blurred vision	Unilateral	Area of paleness with a geographic pattern at the papillomacular bundle	Ellipsoid zone discontinuity	A wedge‐shaped choriocapillaris dropout
MEWDS	F > M	Scotoma/blurred vision	Unilateral	Small white perifoveal dots/mild ant uveitis and vitritis	Abnormal reflectivity of the inner/outer segment line	Normal to small areas of flow reduction at the level of choriocapillaris
AMIC	F = M	Scotoma/ blurred vision	Bilateral	Whitish “placoid” lesions in post pole	Outer retina hyperreflectivity	Patchy choriocapillaris drop‐out
MFC	F > M	Scotoma/blurred vision/floater	Unilateral/bilateral in recurrence	White‐yellow chorioretinal lesions, evolving to punch out scars	Outer retina disruption with sub‐RPE deposits	Patchy choriocapillaris drop‐out
SC	F = M	Scotoma/blurred vision	Bilateral, asymmetric	Grayish peri‐papillary lesions spread centrifugally	Outer retina hyperreflectivity in active lesion/retinal and RPE atrophy in regressed lesions	Geographic area of choriocapillaris drop‐out

Abbreviations: AMIC, acute multifocal ischemic choriocapillaritis; F, female; M, male; MEWDS, multiple evanescent white dot syndrome; MFC, multifocal choroiditis; OCT, optical coherence tomography; OCTA, optical coherence tomography angiography; RPE, retinal pigmented epithelium; SC, serpiginous choroidopathy.

OCTA shows capillary dropout in choriocapillaritis. Considering larger vessel involvement in APMPPE, MFC, and SC, extended areas of choriocapillaris drop‐out are expected. In MEWDS, OCTA appears normal because the endchoriocapillary is involved [1]. Our case demonstrated a wedge‐shaped choriocapillaris dropout, indicating the involvement of larger vessels compared to MEWDS. However, since APMPPE, MFC, and SC are mostly bilateral, unilateral involvement is more compatible with MEWDS.

Posterior pole whitish “placoid” lesions in both eyes are the main clinical manifestation of APMPPE/AMIC. The disease usually manifests as a single episode. Recurrences should be categorized as mixed intermediate forms [1].

Fundus exam in MFC reveals white‐yellow chorioretinal lesions that evolve into punch‐out scars. Recurrent ischemic events and numerous scars are its defining features [1].

Grayish subretinal lesions that originate from the peri‐papillary area and progress centrifugally are characteristic manifestations of serpiginous choroiditis. Active lesions develop into pigmented chorioretinal scars over time [1]. In some patients, the lesion can arise in the macular region [5, 6]. Although our case presented as a single macular choriocapillaris non‐perfusion, the course of the disease and the pattern of progression were not compatible with SC. Compared to AMIC, macular SC shows more retinal pigment epithelial and choroidal atrophy. Patients with macular serpiginous choroiditis are older. The recurrence of lesions is common. Subretinal neovascularization membranes are a usual complication of macular serpiginous choroiditis [5, 6].

Secondary causes have to be considered in patients with inflammatory choriocapillaritis. TB‐related serpiginous‐like choroiditis and acute syphilitic posterior placoid chorioretinitis (ASPPC) are the primary differential diagnoses. Secondary inflammatory choriocapillaritis (SICCP) manifests in different presentations like placoid form with a yellowish lesion in the posterior pole and multiple yellow‐whitish lesions in the posterior pole and mid periphery [7, 8].

Some cases of primary choriocapillaritis present as intermediate forms, which have overlapping symptoms, signs, and clinical courses and cannot be categorized as one of the mentioned PICCPs.

Our case of atypical acute ischemic choriocapillaritis presents distinct clinical features compared to other choriocapillaropathies such as APMPPE, serpiginous choroiditis, MFC, and MEWDS. APMPPE typically manifests with bilateral, multifocal, creamy placoid lesions at the level of the RPE, often following a viral illness, similar to our patient's history of upper respiratory tract infection. However, the unilateral and localized nature of ischemia in our case, as well as the rapid improvement with corticosteroid therapy, is atypical for APMPPE, which usually exhibits a more diffuse involvement of the choriocapillaris. Serpiginous choroiditis, in contrast, tends to be more progressive and recurrent, with extensive geographic areas of chorioretinal atrophy that gradually extend from the optic disc. Our patient's presentation lacks the chronicity and geographic atrophy characteristic of serpiginous lesions. MFC typically affects both eyes, with small, yellowish‐white lesions involving both the posterior pole and periphery, often accompanied by vitreous inflammation—none of which were present in our case. MEWDS, while also often unilateral and affecting young individuals, typically presents with smaller white dots localized in the perifoveal region, and patients often experience a transient photopsia, which our patient did not report. Moreover, MEWDS resolves spontaneously without treatment, unlike our case, where corticosteroid therapy led to clinical improvement. These distinctions highlight the atypical nature of our case and its response to anti‐inflammatory treatment, which may suggest a transient inflammatory or ischemic event rather than a chronic or relapsing disease.

Based on the clinical presentation, course, and OCTA pattern, our patient was diagnosed with atypical APMPPE/AMIC. AMIC typically begins bilaterally; very few unilateral cases have been reported [9, 10, 11]. Unilateral and unifocal involvement make our case unusual regarding the clinical presentation and course of AMIC.

The disease severity is the primary determinant of the treatment indications. Also, Systemic treatment initiation is more likely in patients with atypical presentations [10]. Oral corticosteroid and immunomodulatory agents are used in the more severe conditions, including AMIC, MFC, and SC cases. In contrast, observation is chosen in MEWDS and some milder cases of AMIC [1, 2].

Our patient was treated with oral prednisolone 50 mg/day for a short period (tapered off within 2 weeks). The lesion was entirely resolved.

## Author Contributions

**Mehrdad Motamed Shariati:** conceptualization, investigation, supervision, writing – original draft, writing – review and editing. **Nasser Shoeibi:** conceptualization, supervision, writing – original draft, writing – review and editing. **Mariye Yaghoubi:** data curation, writing – original draft, writing – review and editing.

## Ethics Statement

The authors have nothing to report.

## Consent

Written informed consent was obtained from the patient to publish this case report and any accompanying images. A copy of the written consent is available for review by the Editor‐in‐Chief of this journal.

## Conflicts of Interest

The authors declare no conflicts of interest.

## Data Availability

The datasets used during the current study are available from the corresponding author on reasonable request.
